# Effect of fermentation temperature on the non-volatile components and *in vitro* hypoglycemic activity of Jinxuan black tea

**DOI:** 10.3389/fnut.2024.1498605

**Published:** 2024-11-06

**Authors:** Guangneng Li, Jianyong Zhang, Hongchun Cui, Ying Gao, Debao Niu, Junfeng Yin

**Affiliations:** ^1^National Engineering Research Center for Tea Processing, Key Laboratory of Tea Biology and Resources Utilization, Ministry of Agriculture, Tea Research Institute Chinese Academy of Agricultural Sciences, Hangzhou, China; ^2^College of Light Industry and Food Engineering, Guangxi University, Nanning, China; ^3^Tea Research Institute, Hangzhou Academy of Agricultural Sciences, Hangzhou, China

**Keywords:** fermentation temperature, black tea, non-volatile components, metabolomics, enzyme inhibitory activity, tea pigments

## Abstract

Fermentation significantly influences the chemical composition of black tea, yet the effects of different fermentation temperatures on non-volatile components and their *in vitro* hypoglycemic activity are insufficiently studied. This research investigates how varying temperatures (20, 25, and 30°C) affect the bioactive profile and the inhibitory activity of Jinxuan black tea against α-glucosidase and α-amylase. Our results show that lower fermentation temperatures (20°C) lead to elevated levels of key bioactive compounds, including tea polyphenols (9.24%), soluble sugars (8.24%), thearubigins (7.17%), and theasinesin A (0.15%). These compounds correlate strongly with enhanced α-glucosidase inhibition (R = 0.76–0.97). Non-targeted metabolomic analysis revealed that 36 differential metabolites, including catechins, exhibited altered levels with increasing fermentation temperature. Notably, tea fermented at 20°C exhibited superior hypoglycemic activity, with α-glucosidase inhibition (IC_50_ = 14.00 ± 1.00 μg/ml) significantly outperforming α-amylase inhibition (IC_50_ = 2.48 ± 0.28 mg/ml). The findings of this research underscore the importance of fermentation temperature in optimizing the bioactive profile of black tea. It is proposed that recommendations for future processing or formulation should emphasize the use of lower fermentation temperatures, aimed at augmenting the health benefits linked to higher polyphenol content and stronger hypoglycemic activity.

## Graphical abstract

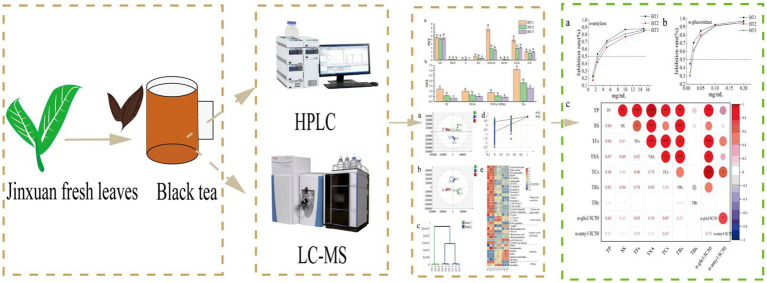

In investigating the effect of fermentation temperature on Jinxuan black tea, it was found that low-temperature fermentation at 20°C enhances bioactivity by preserving key active compounds, including catechins and theaflavins, while also significantly inhibiting digestive enzymes, thereby improving the tea's nutritional and health benefits.

## Highlights

- Fermentation temperature has a significant effect on the active components of Jinxuan black tea.
- Lower temperature (20°C) fermentation retains theaflavins (TFs) and theasinesins (TSs).
- Lower temperature fermentation (20°C) increased the inhibitory activity of α-glucosidase and α-amylase.
- Significant positive correlation between changes in the content of theaflavins and theasinesins and the rate of α-glucosidase inhibition.

## 1 Introduction

Black tea, the most widely consumed tea globally (1), undergoes a unique fermentation process during which catechin-based polyphenols are enzymatically oxidized by polyphenol oxidase (PPO) and peroxidase (POD). This oxidation leads to the gradual formation of theaflavins (TFs), theasinesins (TSs), thearubigins (TRs), and theabrownins (TBs) (2–4). Tea pigments, including TFs, TRs, and TSs, are critical determinants of black tea’s color and taste (5, 6) and exhibit diverse biological activities, including hypoglycemic and hypolipidemic effects (7–9). Theasinesins, particularly theasinesin A (TSA), theasinesin B, and theasinesin C, also significantly influence the color and taste of black tea (2, 10). Furthermore, the inhibition of digestive enzymes and the modulation of blood glucose levels have emerged as significant areas of research in recent years (9, 11, 12).

Diabetes mellitus, a metabolic disorder characterized by hyperglycemia, have detrimental effects on vital organs, including the cardiovascular system and the eyes, and poses a significant risk to life (13). Black tea consumption has been associated with reduced postprandial blood glucose levels, potentially mitigating the risk of type 2 diabetes mellitus (T2DM). This effect is partly due to the fermented components in black tea, which help reduce glucose production in the body (14, 15). Upon ingestion, carbohydrates are degraded by α-amylase in the stomach and small intestine, yielding oligosaccharides such as maltodextrin. Subsequently, α-glucosidase secreted by small intestinal epithelial cells further hydrolyzes these oligosaccharides into glucose, which is then absorbed and utilized. The intake of tea polyphenols, catechins, tea pigments, and other bioactive compounds present in fermented black tea can inhibit the activity of these digestive enzymes, thereby reducing glucose production. This mechanism is considered a promising strategy for controlling postprandial blood glucose levels (14, 15).

Extensive research has been conducted on black tea fermentation technology, exploring the impact of various parameters on the transformation of key bioactive compounds. These parameters include fermentation time (16), fermentation temperature (16–20), fermentation humidity (21), and drying temperature (22). Previous studies have highlighted the influence of fermentation temperature on black tea quality and composition. Obanda et al. (17) found that epicatechin gallate (ECG) and epigallocatechin gallate (EGCG) are the primary catechins remaining after fermentation, leading to increased TFs formation. Samanta et al. (18) demonstrated that a fermentation temperature of 20°C promotes the conversion of catechins to TFs in TV25 tea variety. Qu et al. (19) investigated the effects of varying fermentation temperatures on black tea and concluded that 28°C optimizes both sensory quality and bioactivity. Extreme fermentation temperatures, such as 35°C and 15°C, can negatively impact PPO and POD activity, ultimately affecting black tea quality (19). Zhu et al. (20) observed that a fermentation temperature of 25°C preserved the color of Yunnan big-leaf black tea while promoting the accumulation of TFs and TRs. In contrast, a higher fermentation temperature of 35°C favored the accumulation of TBs. Furthermore, Wang et al. (16) investigated the impact of fermentation time on Yunnan Congou black tea. Their findings indicated that when the fermentation time was 3 h (fermentation at 28°C, 90% relative humidity), catechins were retained in significant quantities, the formation and retention of TFs and TRs were enhanced, and the taste was more favorable at this stage. The results of the aforementioned studies demonstrate that the current research primarily focuses on the impact of fermentation temperature on the conversion of catechins and on flavor quality. Despite significant research on black tea fermentation, investigations exploring the impact of fermentation temperature on *in vitro* hypoglycemic bioactivity and its correlation with specific chemical components remain limited. Notably, the relationship between metabonomic changes of chemical composition and vitro hypoglycemic in black teas fermented to varying degrees deserve further attention.

Fermentation temperature significantly influences the composition and content of bioactive compounds in Jinxuan black tea, thereby affecting its *in vitro* anti-hyperglycemic activity. This process is realized through the regulation of key components (such as catechins, theaflavins, tea polyphenols, and soluble sugars). Additionally, we hypothesize that there is a correlation between the α-glucosidase and α-amylase inhibitory activity and the changes in fermentation temperature, meaning that different fermentation temperatures will lead to varying degrees of enzyme inhibitory activity. In this study, we will conduct experiments at three key fermentation temperatures (20, 25, and 30°C) and utilize non-targeted metabolomics, high-performance liquid chromatography, and colorimetric analysis to explore the changes in black tea metabolites under these temperatures and their relationship with enzyme inhibitory activity. We believe that this hypothesis will provide new insights into understanding how fermentation temperature affects the functional properties of black tea.

## 2 Materials and methods

### 2.1 Chemicals and reagents

Acarbose (Shanghai Dibao Biotechnology Co., Ltd.), p-nitrophenyl-α-D glucopyranoside (p-NPG), α-glucosidase (9001-42-7), and α-amylase (*Bacillus subtilis*, 9000-90-2) were procured from Shanghai Yuanye Biotechnology Co. Tapioca starch (analytically pure) was procured from Bergey (Hubei) Biotechnology Co. The DNS reagents were procured from Beijing Solepol Technology Co. Liquid chromatography–mass spectrometry (LC–MS)-grade formic acid, acetonitrile, and methanol were purchased from Merck (Darmstadt, Germany). Other chemicals and reagents in this study were of analytical.

### 2.2 Preparation of black tea samples with different degrees of fermentation

In July 2023, Jinxuan fresh leaves (one bud and two leaves) were harvested from the test base in Shengzhou, Zhejiang Province. The processing of black tea volved a series of steps, including withering, kneading, fermentation, and drying. The fresh leaves are withered naturally at room temperature (28°C), and then kneaded (Tea Kneading Machine—Model 6CRM-25, Zhejiang Chunjiang Tea Machinery Co.) according to the principle of light-weight and light-weight. Subsequently, the curled leaves were unwound and placed within the fermentation apparatus (intelligent artificial climate chamber, PRX-500D, Ningbo Plante Instrument Co.) for fermentation. The temperature and humidity settings were 20, 25, and 30°C, respectively, with a fermentation time of 5 h. Finally, the leaves were dried uniformly (JY-6CHZ-7B Tea Roaster,Fujian Jiayou Tea Machinery Intelligent Technology Co.), which was recorded as BT1, BT2, and BT3, respectively.

### 2.3 Pre-treatment for chemical composition analysis of black tea samples with different degrees of fermentation

The resulting tea was ground into a uniform powder by a pulverizing grinder (IKA, Germany), and 0.2 g of the tea sample was accurately weighed in a test tube. The sample was then added with 10 ml of boiling water and extracted in a water bath at 100°C. The samples were then incubated for 10 min with shaking every 5 min, after which centrifugation was performed at 8,000 rpm for 10 min (Centrifuge 5810R, Eppendorf, Hamburg, Germany). The resulting supernatant was then filtered through a 0.45 μm filter membrane. Three replicates were conducted for each sample.

### 2.4 Determination of tea polyphenols and tea pigments (TFs-TRs-TBs) in black tea samples

Tea polyphenol content was determined according to folinol method (23), determined with reference to the national standard GB/T 8313-2018. Slight modification of the reference method. A volume of 1 ml of the tea infusion obtained from subsection 2.3 should be transferred to a 100 ml volumetric flask, shaken thoroughly and the volume recorded. A volume of 1 ml should be taken and combined with 5.0 ml of 10% forintol reagent. The solution should be shaken thoroughly and left for a period of 3–8 min. Subsequently, 4.0 ml of 7.5% sodium carbonate solution should be added, and the solution should be shaken once more. The solution should then be left for 60 min to allow for the detection of the absorbance, with pure water serving as the blank control.

Tea pigments (TFs, TRs and TBs) were determined by the method of Hua et al. (2). The sample of ground tea was accurately weighed at 3.00 grams, and then 125 ml of boiling water was added, followed by extraction for 10 min, with shaking every 5 min. Subsequently, the tea extract was obtained by filtration while still at a high temperature. A 100 ml split funnel was filled with 25 ml of ethyl acetate and 25 ml of tea extract, and the mixture was extracted by shaking for 5 min. Layers are separated, the aqueous layer (lower layer) is drained off, and the ethyl acetate (upper layer) is poured out. Pipette 2 ml of the upper solution into a 25 ml volumetric flask and add 95% ethanol to get solution A. Pipette 2 ml of the lower solution, add 2 ml of saturated oxalic acid solution and 6 ml of distilled water, and then add 95% ethanol to volume to 25 ml to obtain solution B. Next, 15 ml of the upper layer and 15 ml of 2.5% sodium bicarbonate solution were added to a 50 ml dispensing funnel and rapidly and strongly shaken for 30 s. After standing and separating, 4 ml of the upper solution was added to 21 ml of 95% ethanol to obtain solution C. Finally, 15 ml of tea extract and 15 ml of n-butanol were pipetted into a 50 ml dispensing funnel, shaken for 3 min, and after standing for layering, 2 ml of the aqueous layer was taken into a 25 ml volumetric flask, and 2 ml of saturated oxalic acid solution and 6 ml of distilled water were sequentially added, and finally, 95% ethanol was added to get solution D. The absorbance of the four solutions was measured at 380 nm. They were noted as EA, EB, EC, and ED, respectively. Theaflavins (%) = 2.25 × EC; thearubigins (%) = 7.06 × (2 EA + 2 EB – EC − 2ED); theabrownins (%) = 2 × ED × 7.06.

### 2.5 Determination of soluble sugars in black tea samples

The soluble sugars were quantified by the anthrone-sulfuric acid colorimetric method, which is analogous to the approach previously described by the subject (24). One milliliter of the sample solution was combined with 4 mL of anthrone sulfuric acid (2 mg/ml), mixed, and incubated in a water bath at 100°C for 10 min. The solution was then cooled to room temperature, and the absorbance at 620 nm was measured.

### 2.6 Analysis of catechins, theaflavins and theasinesin a in black tea samples

According to the method described by Xue et al. (25). the chromatographic column employed was a 5C18-AR-II (4.6 mm × 250 mm). The injection volume was 10 μl, with a detection wavelength of 280 nm. The flow rate was set at 0.8 ml/min, and the column temperature was maintained at 35°C. The mobile phases consisted of A, 50 mmol/L phosphoric acid, and B, 100% acetonitrile. The elution gradients occurred over specific time intervals: from 0 to 39 min, the A-phase decreased from 96 to 70%; from 39 to 54 min, phase A decreased from 70 to 25%; and from 54 to 55 min, phase A increased from 25 to 96%.

### 2.7 LC–MS-based non-targeted metabolomics analysis of black tea samples

The analytical parameters were based on the methodologies outlined by Li (26, 27). The ultra-high performance liquid chromatography (UHPLC-Q-Exactive/MS, Thermo Fisher, San Jose, CA, USA) conditions involved the utilization of a T3 column (100 mm × 2.1 mm, 1.8 μm, Waters, Ireland), with the column temperature set at 40°C and a flow rate of 0.4 ml/min. The injection volume was 3 μl. The mobile phases comprised phase A, which consisted of a 0.1% formic acid aqueous solution, and phase B, containing a 0.1% formic acid in acetonitrile solution. The elution program proceeded as follows: at 0 min, 98% A, 2% B; at 0.5 min, 98% A, 2% B; at 10 min, 85% A, 15% B; at 18 min, 60% A, 40% B; at 20 min, 10% A, 90% B; at 21 min, 98% A, 2% B; and at 25 min, 98% A, 2% B. The mass spectrometry conditions in a quadrupole orbital trap included the use of an electrospray ion source, with a spray ionization source and detection mode set to positive ion mode ESI. The capillary voltage was 3.5 kV, capillary temperature was 300°C, and auxiliary gas temperature and flow rate were 350°C and 10 L/min, respectively. The mass spectrometry scanning range encompassed the mass-to-nucleus ratio (m/z) 100–1,000.

### 2.8 Evaluation of *in vitro* hypoglycemic activity of black tea samples with different degrees of fermentation

#### 2.8.1 *In vitro* inhibition assay of α-glucosidase

The hypoglycemic potential of tea infusion was assessed through *in vitro* inhibition of α-glucosidase as described in the prior work of Li (28). Different concentrations of tea infusion (50 μl) were incubated with α-glucosidase (50 μl, 1 U/ml) with phosphate buffer (pH = 6.8, 0.1 M) at 37°C for 10 min, and then p-NPG (100 μl, 5 mM) was added to react for 15 min, followed by the immediate addition of Na_2_CO_3_ (300 μl, 1 M). Subsequently, the absorbance at 405 nm was quantified utilizing a versatile enzyme marker (CYTATION1, Bio-Tek, Burlington, Vermont, USA). Both sample and blank controls were included, with acarbose serving as the positive control. The inhibition of α-glucosidase was calculated using the formula:


$$\alpha -\mathrm{glucosidase inhibition}\;\left(\%\right)=\left(1-\left(A1-A2\right)/\left(A3-A4\right)\right)\times 100\%$$

Where, A1 and A2 denote the absorbance values of the sample group and the sample blank group, respectively; A3 and A4 denote the absorbance values of the blank group and the blank control, respectively, and the results are expressed as IC_50_ values.

#### 2.8.2 *In vitro* inhibition assay of alpha amylase

The inhibitory effect of tea samples on α-amylase activity was assessed following the protocol established by Li (29). Post-extraction, 0.2 ml of tea infusion at varying concentrations and 0.1 ml of α-amylase (0.5 U/ml) were combined in 2 ml centrifuge tubes and incubated at 25°C for 10 min. Subsequently, 0.2 ml of tapioca starch (10 mg/ml) was introduced and the mixture was further incubated for an additional 10 min. The procedure was concluded by adding 0.4 ml of DNS reagent heated at 100°C for 10 min. Following cooling, 0.1 ml of the reaction solution was transferred into a 96-well enzyme-labeled plate (PBS, 0.1 M, pH 6.9, 6.6 mm NaCl), and the absorbance at 540 nm was quantified using an enzyme marker to calculate α-amylase inhibition.


$$\alpha -\mathrm{amylase inhibition}\;\left(\%\right)=\left(1-\left(A1-A2\right)/\left(A3-A4\right)\right)\times 100\%$$

Where, A1 and A2 denote the absorbance values of the sample group and the sample blank group, respectively; A3 and A4 denote the absorbance values of the blank group and the blank control, respectively, and the results are expressed as IC_50_ values.

### 2.9 Statistical analysis

The experiment was repeated three times for each group and the results were performed as mean ± standard deviation (SD). The results were analyzed by one-way analysis of variance (ANOVA) followed by Duncan *post hoc* test, and IC_50_ was calculated using SPSS 26.0 (SPSS Inc., Chicago, IL, USA) software and considered statistically significant at *p* < 0.05. Heat-map analysis was analysed using Tbtools software. Origin 2021 software (OriginLab, Northampton, MA, USA) was used to draw graphs and correlation analysis.

## 3 Results and discussion

### 3.1 Differences in non-volatile components of black tea at different fermentation temperatures

As illustrated in Table 1, the pertinent non-volatile components in black tea extracts were demonstrably influenced by fermentation temperature. Black tea fermented at 30°C exhibited a pronounced decline in polyphenol and soluble sugar contents in comparison to 20°C. Among these, the contents of TFs, TRs, and TSA decreased significantly, indicating that lower temperature fermentation favors the formation of phenolic pigments.

**Table 1:** Main components of black tea with different fermentation temperatures (%).

Samples	TP	SS	TRs	TBs	TCs	TFs	TSA
BT1	9.24 ± 0.29a	8.24 ± 0.36a	7.14 ± 0.23a	7.36 ± 0.13a	2.19 ± 0.07a	0.31 ± 0.01a	0.15 ± 0.0a
BT2	8.50 ± 0.64a	8.33 ± 0.72a	6.83 ± 0.30a	7.40 ± 0.11a	1.44 ± 0.05b	0.19 ± 0.01b	0.10 ± 0.01b
BT3	7.30 ± 0.29b	7.05 ± 0.72b	5.30 ± 0.19b	7.36 ± 0.16a	1.44 ± 0.06b	0.13 ± 0.00c	0.05 ± 0.00c

TP, tea polyphenols; SS, soluble sugars; TFs, theaflavins; TSA, theasinesin A; TCs, total catechins; TRs, thearubigins; TBs, theabrownins. BT1, BT2, and BT3 indicate fermentation temperatures of 20, 25, and 30°C, respectively. Data are expressed as mean ± standard deviation (*n* = 3). Different lowercase letters in the same column indicate significant differences at the *p* < 0.05 level.

Furthermore, we investigated the changes in catechin and theaflavin monomer profiles across the different fermentation temperatures. These include epicatechin (EC), ECG, epigallocatechin (EGC), EGCG, catechin (C) and gallocatechin (GC).

**Figure 1:**
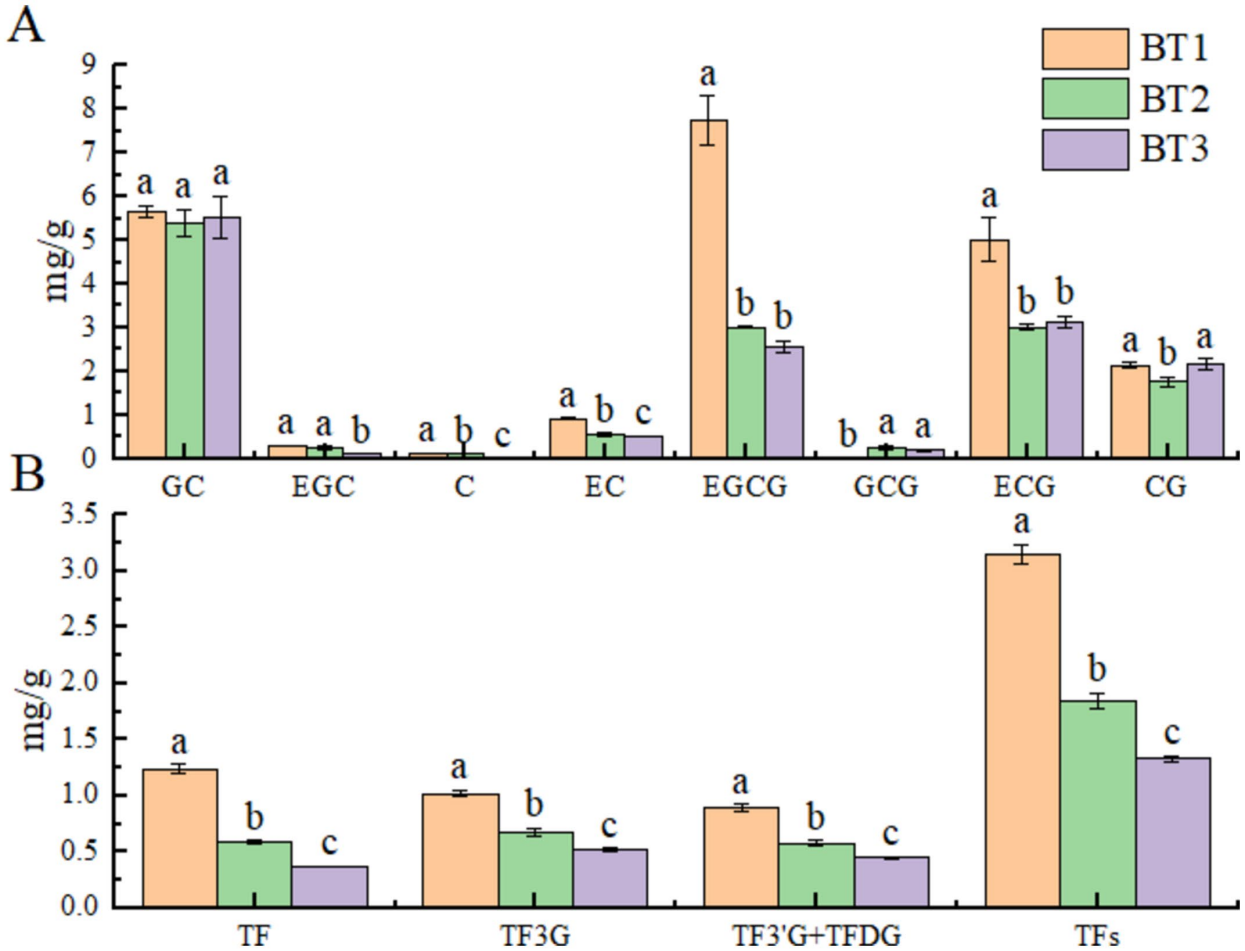
Catechin fraction A and theaflavin fraction B; BT1, BT2, and BT3 indicate fermentation temperatures of 20, 25, and 30°C, respectively. TF, Theaflavin; TF3G, theaflavin-3-gallate; TF3’G, theaflavin-3′-gallate; TFDG, theaflavin-3,3′-digallate.

The contents of catechins and theaflavins following fermentation at varying temperatures for a period of 5 h were presented in Figures 1A,B. Figure 1A illustrated that the contents of various catechins exhibited a distinct variation with respect to fermentation temperature. When the fermentation temperature was set at 25 and 30°C, the EGC, EC content exhibited a notable decline, particularly the EGCG, ECG content. In comparison to BT1, EGCG exhibited a reduction of 61.07 and 66.84%, while ECG demonstrated a decrease of 39.84 and 37.37%. In addition, GC was not sensitive to changes in fermentation temperature. On the contrary, GCG content was increased. The changes in catechin content were found to be affected by both fermentation time and fermentation temperature. A related study demonstrated that ester catechin content was higher than simple catechin at a fermentation time of 5 h (16), which was consistent with the results of this experiment. Figure 1B demonstrated that the theaflavin monomer content of black tea is related to the brightness and flavor of the tea infusion (19). The individual monomeric theaflavin content exhibited a significant variation as a function of fermentation temperature. As the fermentation temperature increased from 20°C to 30°C, a gradual decrease in the total theaflavin content was observed. It has been demonstrated that fermentation at 20°C resulted in higher polyphenol oxidase (PPO) and peroxidase (POD) activities, which led to the formation of a higher theaflavin content (18). In this experiment, the theaflavin substrates (EGC, EC, EGCG, and ECG) were found to be affected by the fermentation temperature. Fermentation temperature of 20°C was observed to retain more EGCG and ECG, which was in agreement with the experimental results of Obanda (17). Furthermore, the levels of ECG and EGCG were negatively correlated with α-glucosidase activity, as previously observed (30), and this relationship was also confirmed in the present experiment (Table 2). It has been demonstrated that the conversion of TFs to more TRs occurs at higher temperatures, in contrast to lower fermentation temperature of 20°C (18). In contrast, the TRs content of the sample with a fermentation temperature of 30°C was found to be lower than that of the sample with a fermentation temperature of 20°C. This finding was at odds with the results of previous studies (2, 19), which may be attributed to differences in the varieties of fresh leaf raw material, enzyme activity, fermentation duration, and other factors. This is due to the fact that disparate fermentation temperatures may impact the activity of enzymes linked to tea processing (e.g., PPO and POD) (18), which in turn influences the production and conversion of non-volatile components. At the same time, it is important to recognize that the fermentation process may change dynamically over time, despite the fixed 5-h fermentation duration that was examined in our study. Different fermentation durations may result in different profiles of bioactive compounds and enzyme activities. To better capture these dynamic changes, future studies should explore a broader range of fermentation durations.

**Table 2:** Effects of black tea with different fermentation temperatures on the inhibitory activities of α-amylase and α-glucosidase.

Samples	IC_50_ α-Amylase (mg/ml)	IC_50_ α-Glucosidase (μg/ml)
BT1	2.48 ± 0.28b	14.00 ± 1.00b
BT2	3.64 ± 0.06a	19.33 ± 1.15a
BT3	2.92 ± 0.51ab	19.33 ± 1.53a
Acarbose	0.041 ± 0.00	0.29 ± 0.028

BT1, BT2, and BT3 indicate fermentation temperatures of 20, 25, and 30°C, respectively. Data are expressed as mean ± standard deviation (*n* = 3). Different lowercase letters in the same column indicate significant differences at the *p* < 0.05 level.

### 3.2 Non-targeted metabolomics analysis based on LC–MS

**Figure 2:**
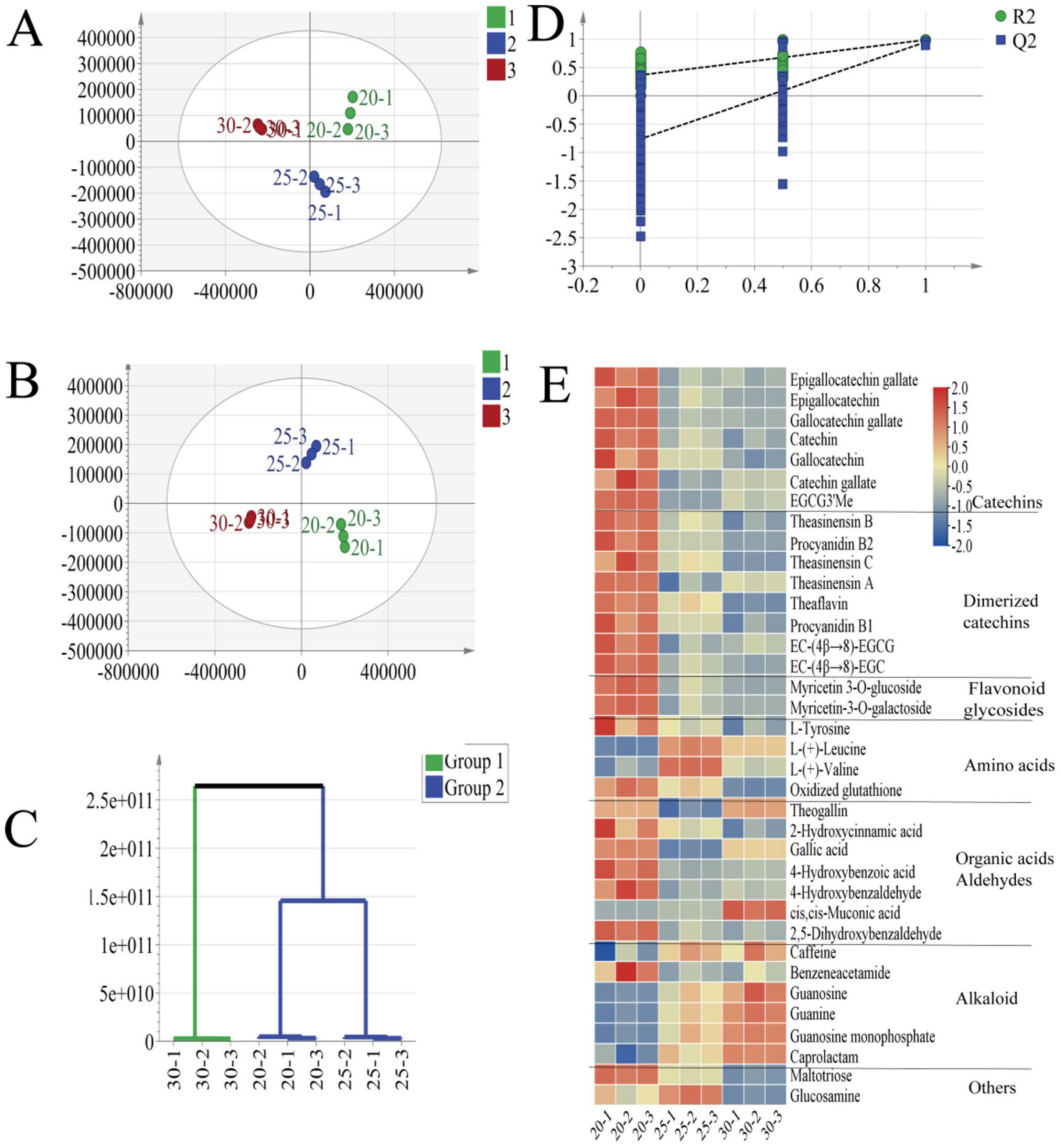
Plots of PCA scores (A) and OPLS-DA scores (B) of three black teas at different fermentation temperatures; results of HCA analysis of the OPLS-DA model (C); cross-validation plots obtained from 200 perturbation tests (D); and heatmap analysis of 36 differential compounds after normalization (E).

After peak matching and calibration using the Chemspider, mzcloud, and mzvault databases, a total of 2,706 compound ions were identified. These metabolites were further analyzed by PCA and OPLS-DA modeling using Simca software (Version 14.1). PCA analysis (Figure 2A) showed that all samples were presented with 95% confidence intervals, which proved the stability and reliability of the overall imported data. To further investigate the key compounds responsible for the differences between groups of tea samples with different fermentation temperatures, the supervised OPLS-DA method was used for statistical analysis. In both PCA and OPLS-DA models, there was a clear trend of separation between samples, indicating significant differences in metabolite compositions at different fermentation temperatures. Figure 2B shows the OPLS-DA validation model, and the intercepts of R^2^ and Q^2^ for the cross-validation model (Figure 2C) were 0.365 and −0.762, respectively, indicating that the OPLS-DA model was reliable. In addition, hierarchical cluster analysis (HCA) showed that the three fermentation temperatures were categorized into two groups, 20°C-25°C as one group and 30°C as one group, and this result indicated that when the fermentation temperature was 30°C may lead to compositional differences. Tentative identification of non-volatiles was based on comparing retention time, m/z values, MS/MS fragments and references (26, 27) with standards or data from databases (e.g., Massbank and MzCloud) when standards were unavailable. All metabolites were then screened for a VIP > 1 and *p* < 0.05, resulting in the identification of 36 significantly different compounds (Supplementary Table S1). These included catechins, dimerized catechins, amino acids, flavonoid glycosides, phenolic acids, and alkaloids, as shown in Figure 2E.

#### 3.2.1 Metabolomic analysis of catechins

Catechins, being the predominant polyphenol compounds in tea, make up approximately 60 to 80% of all tea polyphenols and are classified as flavanols. Extensively studied for their pharmacological effects, catechins are known for their antioxidant and antihyperglycemic properties (31). Among the catechin fractions identified in the non-targeting analysis, the trends of EGCG, EGC, and C exhibited consistency with the results of the quantitative analysis (Figures 1, 2). However, the trends of GCG, GC, and CG were found to be distinct. All three catechin fractions exhibited a declining pattern as the fermentation temperature was elevated from 20 to 30°C. This observation suggests that the fermentation temperature exerts a more pronounced influence on the alterations of catechins. The diverse effects and pathways of oxidative polymerization, cleavage, and isomerization of catechins under varying fermentation temperature treatment conditions exhibited disparate effects on the composition and content of the final formed TSs, TFs, and TRs (32–34).

#### 3.2.2 Metabolomic analysis of dimerized catechins

In this study, eight dimerized catechins were identified. As the fermentation temperature was elevated from 20°C to 30°C, a number of compounds exhibited a declining trend, including epigallocatechin-4β-8-epigallocatechin gallate, theasinesin B, proanthocyanidin B2, theasinesin C, TSA, theaflavin, proanthocyanidin B1, and epigallocatechin-4β-8-epigallocatechin gallate. Among the identified compounds, the trends of theaflavin and TSA were consistent with the results of the targeting analysis, and they were also the key bioactive components in black tea. TFs and TSs are competing products of catechins, which are partly oxidized to TFs and partly reduced to TSs during black tea processing, and finally TFs and TSs are oxidized by coupling to produce TRs (35, 36). It has been shown that high PPO activity during fermentation at lower temperatures leads to the gradual oxidation of various catechins to TFs and TSs (2, 37). In addition to this, fermentation time also plays a crucial role in the formation of TFs and TRs in black tea (16).

#### 3.2.3 Amino acid metabolomic analysis

Amino acids, major chemical components of tea, contribute significantly to its fresh flavor profile, imparting a crisp and refreshing character to the tea infusion. Following exposure to various fermentation temperatures, tyrosine, leucine, valine, and glutathione were discerned as distinctive compounds.

The heatmap illustrated a decline in tyrosine and glutathione levels as fermentation temperature rose, whereas leucine and valine exhibited an initial rise followed by a decline with escalating fermentation temperature. This phenomenon arises from protein breakdown during the pre-fermentation phase, resulting in a progressive buildup of amino acids. Subsequently, upon completion of fermentation, certain amino acids partake in biochemical processes within tea, generating aromatic compounds and pigments characteristic of black tea (38). In addition, high temperatures promote the conversion of amino acids to secondary metabolites, resulting in lower amino acid content (19).

#### 3.2.4 Flavonoid glycosides metabolomic analysis

Flavonoid glycosides are pivotal in imparting astringency to tea infusion. Elevated levels of flavonoid glycosides are detrimental to the development of premium tea flavor and notably contribute to the intensification of caffeine bitterness (39, 40). Both populin-3-O-galactoside and populin-3-O-glucoside exhibited a decrease in concentration with an increase in fermentation temperature. It has been demonstrated that the concentration of these substances was highest at a fermentation time of 4 h (fermentation at 28°C), as a result of the formation of different flavonoid glycosides under varying conditions (16).

### 3.3 Hypoglycemic activity analysis

#### 3.3.1 Analysis of alpha-glucosidase and alpha-amylase inhibition

In this study, the inhibitory effects on digestive enzymes of black tea produced at different fermentation temperatures were investigated. According to the findings presented in Table 2, black tea fermented at a lower temperature (20°C) exhibited superior digestive enzyme inhibitory activity. Numerous studies have indicated that catechins and TFs in tea possess potent digestive enzyme inhibitory effects, and that low-temperature fermentation enhances the activity of PPO, thereby promoting the formation of TFs and TSs (2). Conventional compositional tests also revealed that black tea fermented at a low temperature (20°C) had the highest theaflavin and TSA content. Research has shown that TSA demonstrates better inhibitory effects than EGCG in inhibiting α-glucosidase, with TSA containing more hydroxyl and gallic groups in its structure (11). However, there is limited literature available on the inhibition of α-amylase activity by TSA. In this experiment, tea infusion inhibited α-glucosidase better than α-amylase, with IC_50_ of 14.0 μg/ml and 2.48 mg/ml, which were higher than that of acarbose by 0.29 μg/ml when the fermentation temperature was 20°C. When the fermentation temperature was raised to 25°C, the inhibitory activity against α-amylase significantly decreased, aligning with the changes observed in catechins, dimerized catechins, and flavonoid glycosides. Furthermore, macromolecular tea polysaccharides were found to possess inhibitory effects on digestive enzymes. Specifically, a 67.1% inhibition of α-glucosidase was observed at a concentration of 1.0 mg/ml of black tea polysaccharides, attributed to the high content of monosaccharides and proteins in black tea polysaccharides, which synergistically enhanced the inhibition of α-glucosidase (41). Among the 36 identified differential compounds, most catechins, dimeric catechins, and flavonoid glycosides were reported to exhibit inhibitory effects on digestive enzymes (42). Consequently, maintaining the fermentation at a lower temperature facilitates the retention of catechins and the gradual formation of dimerized catechins, thereby enhancing the potential hypoglycemic effect of black tea.

### 3.4 Correlation analysis of active ingredients with digestive enzymes

**Figure 3:**
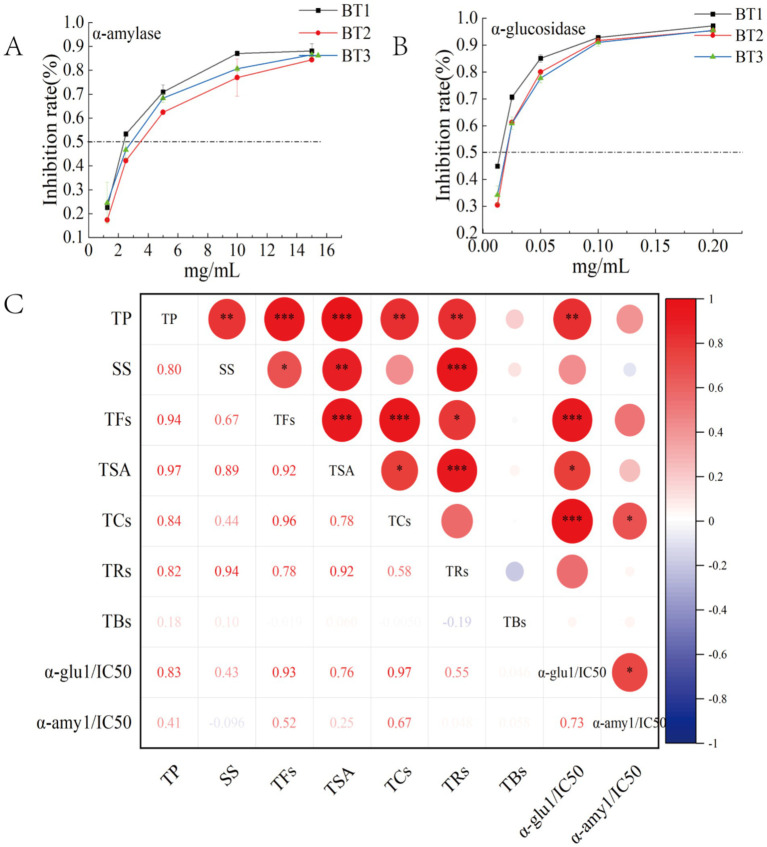
Heat map of Pearson ‘s correlation analysis of α-amylase (A), α-glucosidase inhibitory activity (B), and digestive enzyme inhibitory activity with the main constituents of black tea at different fermentation temperatures (C); BT1, BT2, and BT3 denote the fermentation temperatures of 20, 25, and 30°C, respectively; *p < 0.05, **p < 0.01 and ***p < 0.001. TCs, total catechins.

In order to establish a more robust correlation between the primary bioactive constituents and digestive enzymes present in black tea subjected to varying fermentation temperatures, a Pearson correlation analysis was conducted. The inhibitory rate was represented as 1/IC_50_, where a larger 1/IC_50_ value corresponds to a more potent inhibitory effect. This is illustrated in Figure 3.

Figure 3 and Supplementary Table S2 depict the correlation between metabolites and 1/IC_50_ α-glucosidase values. Four common physicochemical constituents, along with 26 non-targeted metabolites, exhibited a significant positive correlation with the rate of α-glucosidase inhibition at the *p* < 0.01 or *p* < 0.05 level. The four common physicochemical constituents, namely tea polyphenols, soluble sugars, TFs, and total catechins (TCs), demonstrated correlations (R ranging from 0.76 to 0.97). Additionally, the 26 non-targeted metabolites, including 7 catechins (R ranging from 0.86 to 0.97), 8 dimerized catechins (R ranging from 0.83 to 0.96), 2 flavonoids (R ranging from 0.96 to 0.97), 2 amino acids (R ranging from 0.76 to 0.82), 5 organic acids and aldehydes (R ranging from 0.73 to 0.98), benzeneacetamide, and maltotriose (R ranging from 0.88 to 0.89), exhibited significant correlations. These findings suggest that these compounds may exert potent inhibitory effects on α-glucosidase. The data presented in Figure 2E illustrates a substantial reduction in the levels of catechins, dimeric catechins, and flavonoids as the fermentation temperature rises, potentially contributing to the diminished inhibition of α-glucosidase. Likewise, the metabolites exhibiting 1/IC_50_ α-amylase values indicated that a single common physicochemical constituent (total catechins, R of 0.67) and 10 non-targeted metabolites (three catechins, R ranging from 0.68 to 0.69; three dimerized catechins, R ranging from 0.73 to 0.83; and four organic acids and aldehydes, R ranging from 0.73 to 0.81) displayed a significant positive correlation with the rate of α-amylase inhibition. These findings suggest that these compounds may exert effective inhibitory effects on α-amylase. Furthermore, this was corroborated by Figures 3A,B, demonstrating that low-temperature fermentation (20°C) yielded the most potent digestive enzyme inhibitory activity.

Previous research has demonstrated that TFs and TRs, extracted from black tea, lowered plasma glucose levels in rats fed a high-sugar and high-fat diet (7). This effect could be attributed to the ability of TFs and TRs to suppress glucose production within the body by inhibiting α-glucosidase activity, consequently decreasing the influx of glucose into the bloodstream and lowering blood glucose levels. Moreover, TSA has been shown to enhance the secretion of insulin and glucagon-like peptide 1, activate the IRS/PI3K/AKT/GLUT2 signaling pathway in the liver, and modulate intestinal microbiota to ameliorate hyperglycemia in a diabetic mouse model (12). Recent studies have demonstrated that TFs, which are present in black tea extracts, have remarkable inhibitory effects on α-amylase activity (8, 43). This is primarily attributed to the presence of a galloyl structure in their molecular structure, which enhances binding to α-amylase, thereby increasing their inhibitory activity on the enzyme (44). The application of different drying temperatures affects the molecular conformation. For instance, an increase in drying temperature promotes the conversion of EGCG to GCG, which in turn influences the capacity of these compounds to bind to enzymes and thus their inhibitory activity (45). However, further research is needed to determine how the conformation of bioactive components, such as phenolic compounds and catechins, changes in response to variations in fermentation temperature. Additionally, changes in the concentration of distinct bioactive compounds result in fluctuations in the fluorescence intensity of digestive enzymes. These concentration changes affect the interaction forces between molecules and proteins, including hydrogen bonding and hydrophobic interactions, which subsequently impact the activity of the involved enzymes (8, 43–45). Furthermore, the hypoglycemic effects of plant polysaccharides have been extensively documented. Tea polysaccharides derived from teas undergoing various fermentation levels exhibit improved inhibitory activity against digestive enzymes. Moreover, the bioactivity of these polysaccharides is heightened as the fermentation degree increases, with lower molecular weight tea polysaccharides demonstrating superior hypoglycemic effects (41, 46, 47). It is evident that further in-depth studies on the mechanism of *in vitro* hypoglycemic activity of black teas with different degrees of fermentation are required, with particular focus on TSs.

## 4 Conclusion

Fermentation temperature significantly influences the bioactive composition of black tea. A fermentation temperature of 20°C demonstrated enhanced biological activity, preserving higher levels of key bioactive compounds such as catechins (EGCG, ECG), TFs, and TSA. This resulted in a more potent inhibition of digestive enzymes. Our findings demonstrate that lower fermentation temperatures (20°C) promote the preservation of bioactive components in black tea, with their content directly correlated to the inhibition of digestive enzymes. It is important to note that this study only considered *in vitro* enzyme inhibition. Future studies will investigate the intrinsic mechanisms by which these biologically active compounds (e.g., TSA) inhibit digestive enzyme activity and whether they have the same *in vivo* inhibitory effect.

## Data Availability

The original contributions presented in the study are included in the article/Supplementary material, further inquiries can be directed to the corresponding authors.

## References

[1] Pan R Xu Y Liu L You X Chen F Leng Y. A brief analysis of tea import and export trade structure in China during 2022. China Tea. (2023) 45:31–5.

[2] Hua J Wang H Yuan H Yin P Wang J Guo G. New insights into the effect of fermentation temperature and duration on catechins conversion and formation of tea pigments and theasinensins in black tea. J Sci Food Agric. (2022) 102:2750–60. doi: 10.1002/jsfa.11616, PMID: 34719036

[3] Lee L-S Kim Y-C Park J-D Kim Y-B Kim S-H. Changes in major polyphenolic compounds of tea (*Camellia sinensis*) leaves during the production of black tea. Food Sci Biotechnol. (2016) 25:1523–7. doi: 10.1007/s10068-016-0236-y, PMID: 30263440 PMC6049246

[4] Jin G Wang Y Li M Li T Huang W Li L. Rapid and real-time detection of black tea fermentation quality by using an inexpensive data fusion system. Food Chem. (2021) 358:129815. doi: 10.1016/j.foodchem.2021.129815, PMID: 33915424

[5] Jiang Y Hua J Wang B Yuan H Ma H. Effects of variety, season, and region on Theaflavins content of fermented Chinese congou black tea. J Food Qual. (2018) 2018:e5427302:1–9. doi: 10.1155/2018/5427302

[6] Ghosh A Tamuly P Bhattacharyya N Tudu B Gogoi N Bandyopadhyay R. Estimation of theaflavin content in black tea using electronic tongue. J Food Eng. (2012) 110:71–9. doi: 10.1016/j.jfoodeng.2011.12.007

[7] Imran A Butt MS Arshad MS Arshad MU Saeed F Sohaib M. Exploring the potential of black tea based flavonoids against hyperlipidemia related disorders. Lipids Health Dis. (2018) 17:57. doi: 10.1186/s12944-018-0688-6, PMID: 29592809 PMC5872535

[8] Li M Dong Y Kang M Tao T Li W Zhang S. Potential anti-hyperglycemic activity of black tea theaflavins through inhibiting α-amylase. Food Chem. (2024) 22:101296. doi: 10.1016/j.fochx.2024.101296, PMID: 38550892 PMC10972827

[9] Miyata Y Tamaru S Tanaka T Tamaya K Matsui T Nagata Y. Theflavins and Theasinensin a derived from fermented tea have Antihyperglycemic and Hypotriacylglycerolemic effects in KK-ay mice and Sprague–Dawley rats. J Agric Food Chem. (2013) 61:9366–72. doi: 10.1021/jf400123y, PMID: 24011231

[10] Zhang J Cui H Feng Z Wang W Zhao Y Deng Y. Bitterness quantification and simulated taste mechanism of theasinensin a from tea. Front Nutr. (2023) 10:1138023. doi: 10.3389/fnut.2023.1138023, PMID: 37229471 PMC10203438

[11] Tao S Chen G Xu W Peng Y Wan P Sun Y. Preparation of theasinensin a and theasinensin B and exploration of their inhibitory mechanism on α-glucosidase. Food Funct. (2020) 11:3527–38. doi: 10.1039/C9FO03054A, PMID: 32255112

[12] Xu W Huang Y Zhou W Peng Y Kan X Dong W. Theasinensin a attenuated diabetic development by restoring glucose homeostasis, improving hepatic steatosis and modulating gut microbiota in high-fat-diet/streptozotocin-induced diabetic mice. Food Sci Human Wellness. (2023) 12:2073–86. doi: 10.1016/j.fshw.2023.03.026

[13] Constantino MI Molyneaux L Limacher-Gisler F Al-Saeed A Luo C Wu T. Long-term complications and mortality in young-onset diabetes: type 2 diabetes is more hazardous and lethal than type 1 diabetes. Diabetes Care. (2013) 36:3863–9. doi: 10.2337/dc12-2455, PMID: 23846814 PMC3836093

[14] Gao J Chen D Lin Z Peng J Yu S Zhou C. Research progress on the antidiabetic activities of tea and its bioactive components. Beverage Plant Res. (2023) 3:32. doi: 10.48130/BPR-2023-0032

[15] Li G Zhang J Cui H Feng Z Gao Y Wang Y. Research Progress on the effect and mechanism of tea products with different fermentation degrees in regulating type 2 diabetes mellitus. Food Secur. (2024) 13:221. doi: 10.3390/foods13020221, PMID: 38254521 PMC10814445

[16] Wang H Shen S Wang J Jiang Y Li J Yang Y. Novel insight into the effect of fermentation time on quality of Yunnan congou black tea. LWT. (2022) 155:112939. doi: 10.1016/j.lwt.2021.112939

[17] Obanda M Okinda Owuor P Mang’oka R. Changes in the chemical and sensory quality parameters of black tea due to variations of fermentation time and temperature. Food Chem. (2001) 75:395–404. doi: 10.1016/S0308-8146(01)00223-0

[18] Samanta T Cheeni V Das S Roy AB Ghosh BC Mitra A. Assessing biochemical changes during standardization of fermentation time and temperature for manufacturing quality black tea. J Food Sci Technol. (2015) 52:2387–93. doi: 10.1007/s13197-013-1230-5, PMID: 25825546 PMC4375181

[19] Qu F Zeng W Tong X Feng W Chen Y Ni D. The new insight into the influence of fermentation temperature on quality and bioactivities of black tea. LWT. (2020) 117:108646. doi: 10.1016/j.lwt.2019.108646

[20] Zhu J Wang J Yuan H Ouyang W Li J Hua J. Effects of fermentation temperature and time on the color attributes and tea pigments of Yunnan congou black tea. Food Secur. (2022) 11:1845. doi: 10.3390/foods11131845, PMID: 35804663 PMC9265920

[21] Zhang S Jiang X Li C Qiu L Chen Y Yu Z. Effect of fermentation humidity on quality of congou black tea. Food Secur. (2023) 12:1726. doi: 10.3390/foods12081726, PMID: 37107521 PMC10138149

[22] Su S Long P Zhang Q Wen M Han Z Zhou F. Chemical, sensory and biological variations of black tea under different drying temperatures. Food Chem. (2024) 446:138827. doi: 10.1016/j.foodchem.2024.138827, PMID: 38402772

[23] Gao Y Wang J-Q Fu Y-Q Yin J-F Shi J Xu Y-Q. Chemical composition, sensory properties and bioactivities of *Castanopsis lamontii* buds and mature leaves. Food Chem. (2020) 316:126370. doi: 10.1016/j.foodchem.2020.126370, PMID: 32062229

[24] Gao Y Cao Q-Q Chen Y-H Granato D Wang J-Q Yin J-F. Effects of the baking process on the chemical composition, sensory quality, and bioactivity of Tieguanyin oolong tea. Front Nutr. (2022) 9:881865. doi: 10.3389/fnut.2022.881865, PMID: 35651510 PMC9150783

[25] Xue J Jiang H Long D Wang W Zhang J. Simultaneous multiresidue determination od thessinensins and theaflavins in tea useing HPLC. J Chin Inst Food Sci Tech. (2014) 14:237–43. doi: 10.16429/j.1009-7848.2014.05.006

[26] Li Y Zhou H Tian T Hou Y Chen D Zhou J. Nontargeted and targeted metabolomics analysis for evaluating the effect of “golden flora” amount on the sensory quality, metabolites, and the alpha-amylase and lipase inhibitory activities of Fu brick tea. Food Chem. (2023) 416:135795. doi: 10.1016/j.foodchem.2023.135795, PMID: 36871505

[27] Gao J Zhou M Chen D Xu J Wang Z Peng J. High-throughput screening and investigation of the inhibitory mechanism of α-glucosidase inhibitors in teas using an affinity selection-mass spectrometry method. Food Chem. (2023) 422:136179. doi: 10.1016/j.foodchem.2023.136179, PMID: 37119598

[28] Li S Wang R Hu X Li C Wang L. Bio-affinity ultra-filtration combined with HPLC-ESI-qTOF-MS/MS for screening potential α-glucosidase inhibitors from *Cerasus humilis* (Bge.) Sok. Leaf-tea and *in silico* analysis. Food Chem. (2022) 373:131528. doi: 10.1016/j.foodchem.2021.131528, PMID: 34774376

[29] Li F Luo T Hou J Fei T Zhang J Wang L. Natural α-glucosidase and α-amylase inhibitors from raspberry (*Rubus corchorifolius* L.) leaf-tea: screening, identification and molecular docking analysis. LWT. (2023) 181:114763. doi: 10.1016/j.lwt.2023.114763

[30] Lee K-W Na I-S Lee D-H Kim J-W Lee N-Y Chung J-O. Exploratory data analysis of the correlation between bioactive components in various tea extracts and the digestive function *in vitro*. Food Biosci. (2024) 60:104404. doi: 10.1016/j.fbio.2024.104404

[31] Wan C Ouyang J Li M Rengasamy KRR Liu Z. Effects of green tea polyphenol extract and epigallocatechin-3-O-gallate on diabetes mellitus and diabetic complications: recent advances. Crit Rev Food Sci Nutr. (2022) 64:5719–47. doi: 10.1080/10408398.2022.2157372, PMID: 36533409

[32] Fang J Sureda A Silva AS Khan F Xu S Nabavi SM. Trends of tea in cardiovascular health and disease: a critical review. Trends Food Sci Technol. (2019) 88:385–96. doi: 10.1016/j.tifs.2019.04.001

[33] Long P Rakariyatham K Ho C-T Zhang L. Thearubigins: formation, structure, health benefit and sensory property. Trends Food Sci Technol. (2023) 133:37–48. doi: 10.1016/j.tifs.2023.01.013

[34] Wang S Zeng T Zhao S Zhu Y Feng C Zhan J. Multifunctional health-promoting effects of oolong tea and its products. Food Sci Human Wellness. (2022) 11:512–23. doi: 10.1016/j.fshw.2021.12.009

[35] Lin Z Lv H Zhang S. Chemical and pharmacological effects of active components in tea. China Tea. (2018) 40:1–6.

[36] Xu B Xue J Jiang H-Y Zhang J Wang Y. Review on Theasinensins in tea. J Tea Sci. (2014) 34:315–23. doi: 10.13305/j.cnki.jts.2014.04.001

[37] Hua J Yuan H Yao Y Jiang Y Wang J. Effect of temperature on color and tea pigment content of fermented tea leaves. Trans Chin Soc Agric Eng. (2018) 34:300–8. doi: 10.11975/j.issn.1002-6819.2018.12.038

[38] Wan X. Tea biochemistry. Beijing: China Agriculture Press (2003). 451.

[39] Zhong Q Peng J Dai W Lin Z Lv H Chen Q. Analysis of differences in chemical constituents of Rougui rock tea with different roasting degrees by ultra-high performance liquid chromatography-Quadrupole Orbitrap mass spectrometry. Food Sci. (2023) 44:268–82. doi: 10.7506/spkx1002-6630-20221205-052

[40] Wang H Hua J Jiang Y Wang J Yuan H. Effect of different heat transfer modes during secondary drying on quality components, color and taste of congou black tea. Food Sci. (2020) 41:148–57. doi: 10.7506/spkx1002-6630-20190626-349

[41] Xiang G Sun H Chen Y Guo H Liu Y Li Y. Antioxidant and hypoglycemic activity of tea polysaccharides with different degrees of fermentation. Int J Biol Macromol. (2023) 228:224–33. doi: 10.1016/j.ijbiomac.2022.12.114, PMID: 36529215

[42] Li N Zhu H-T Wang D Zhang M Yang C-R Zhang Y-J. New Flavoalkaloids with potent α-Glucosidase and Acetylcholinesterase inhibitory activities from Yunnan black tea ‘Jin-Ya. J Agric Food Chem. (2020) 68:7955–63. doi: 10.1021/acs.jafc.0c02401, PMID: 32628847

[43] Wang Z Gao J Zhao Y Zhou M Chen D Zhou C. High-throughput screening, “protein–metabolite” interaction, and hypoglycemic effect investigations of α-amylase inhibitors in teas using an affinity selection-mass spectrometry method. LWT. (2024) 203:116392. doi: 10.1016/j.lwt.2024.116392

[44] Sun L Song Y Chen Y Ma Y Fu M Liu X. The galloyl moiety enhances the inhibitory activity of catechins and theaflavins against α-glucosidase by increasing the polyphenol–enzyme binding interactions. Food Funct. (2021) 12:215–29. doi: 10.1039/D0FO02689A, PMID: 33295908

[45] Zhou J Zhang L Meng Q Wang Y Long P Ho C-T. Roasting improves the hypoglycemic effects of a large-leaf yellow tea infusion by enhancing the levels of epimerized catechins that inhibit α-glucosidase. Food Funct. (2018) 9:5162–8. doi: 10.1039/C8FO01429A, PMID: 30246823

[46] Xu L Chen Y Chen Z Gao X Wang C Panichayupakaranant P. Ultrafiltration isolation, physicochemical characterization, and antidiabetic activities analysis of polysaccharides from green tea, oolong tea, and black tea. J Food Sci. (2020) 85:4025–32. doi: 10.1111/1750-3841.15485, PMID: 33037621

[47] Xia B Liu Q Sun D Wang Y Wang W Liu D. Ultrasound-assisted deep eutectic solvent extraction of polysaccharides from Anji white tea: characterization and comparison with the conventional method. Food Secur. (2023) 12:588. doi: 10.3390/foods12030588, PMID: 36766120 PMC9914869

