# Supplementary material for: Effect of fermentation temperature on the non-volatile components and in vitro hypoglycemic activity of Jinxuan black tea
Source: Front Nutr. 2024 Nov 6;11:1498605. doi: 10.3389/fnut.2024.1498605 (PMC11576308; doi:10.3389/fnut.2024.1498605)
Supplement: Supplementary file 1 [file Table_1.DOCX]

Effect of fermentation temperature on the non-volatile components and in vitro hypoglycemic activity of Jinxuan black tea

Guangneng Li^a,b,1^, Jianyong Zhang^a,1^, Hongchun Cui^c^, Ying Gao^a^, Debao Niu^b*^, Junfeng Yin^a*^

^a^ Tea Research Institute Chinese Academy of Agricultural Sciences, National Engineering Research Center for Tea Processing, Key Laboratory of Tea Biology and Resources Utilization, Ministry of Agriculture, Hangzhou, China

^b^ College of Light Industry and Food Engineering, Guangxi University, Nanning 530003, China

^c^ Tea Research Institute, Hangzhou Academy of Agricultural Sciences, Hangzhou 310024, China

______________________

*Corresponding author. *E-mail address*: [happyndb@gxu.edu.cn](mailto:happyndb@gxu.edu.cn) (Debao Niu); [yinjf@tricaas.com](mailto:yinjf@tricaas.com) (Jun-feng Yin)

^1^ Guangneng Li, Jianyong Zhang contributed equally to this work.

**Table S1**. Metabolites with VIP values > 1 and *p*＜0.05 for heat map

| Primary ID. | RT (min) | Accurate mass (m/z) | Compounds | VIP | *p* |
| --- | --- | --- | --- | --- | --- |
|  |  |  | **Catechins** |  |  |
| 17 | 10.092 | 459.0926 | EGCG | 5.30 | 0.001 |
| 41 | 7.25 | 307.0813 | EGC | 4.22 | 0.003 |
| 137 | 11.048 | 459.0926 | GCG | 2.52 | 0.000 |
| 152 | 7.706 | 291.0865 | C | 1.77 | 0.010 |
| 146 | 4.662 | 307.0814 | GC | 2.34 | 0.001 |
| 372 | 13.224 | 443.0977 | CG | 1.14 | 0.003 |
| 545 | 12.322 | 473.1083 | Epigallocatechin 3-O-(3-O-methyl)gallate | 1.25 | 0.000 |
|  |  |  | **Dimeric catechins** |  |  |
| 25 | 1.935 | 165.0548 | EC-(4β→8)-EGC | 1.99 | 0.044 |
| 75 | 6.336 | 763.1511 | Theasinensin B | 3.12 | 0.001 |
| 106 | 8.85 | 579.1502 | Procyanidin B2 | 2.50 | 0.001 |
| 119 | 5.556 | 611.1400 | Theasinensin C | 2.73 | 0.017 |
| 154 | 8.958 | 915.1625 | Theasinensin A | 1.60 | 0.001 |
| 258 | 16.216 | 565.1347 | Theaflavin | 1.66 | 0.002 |
| 313 | 8.703 | 579.1502 | Procyanidin B1 | 1.27 | 0.003 |
| 422 | 9.857 | 747.1561 | EC-(4β→8)-EGCG | 1.22 | 0.002 |
|  |  |  | **Flavonol glycosides and flavone glycosides** |  |  |
| 178 | 12.093 | 481.0990 | Myricetin 3-O-glucoside | 5.64 | 0.000 |
| 227 | 11.897 | 481.0991 | Myricetin-3-O-galactoside | 2.60 | 0.000 |
|  |  |  | **Amino acids** |  |  |
| 30 | 1.961 | 182.0813 | L-Tyrosine | 1.96 | 0.038 |
| 64 | 2.132 | 132.1020 | L-(+)-Leucine | 5.59 | 0.000 |
| 99 | 1.171 | 118.0863 | L-(+)-Valine | 2.53 | 0.000 |
| 121 | 1.742 | 613.1599 | Oxidized glutathione | 1.93 | 0.014 |
|  |  |  | **Orgnic acids and aldehydes** |  |  |
| 13 | 3.149 | 345.0817 | Theogallin | 5.64 | 0.000 |
| 83 | 3.149 | 171.0289 | Gallic acid | 2.60 | 0.000 |
| 25 | 1.935 | 165.0548 | 2-Hydroxycinnamic acid | 1.99 | 0.044 |
| 110 | 10.139 | 139.0390 | 4-Hydroxybenzoic acid | 2.49 | 0.000 |
| 156 | 13.032 | 123.0442 | 4-Hydroxybenzaldehyde | 1.56 | 0.002 |
| 170 | 4.833 | 143.0340 | cis,cis-Muconic acid | 2.90 | 0.000 |
| 328 | 7.322 | 139.0391 | 2,5-Dihydroxybenzaldehyde | 1.47 | 0.000 |
|  |  |  | **Alkaloids** |  |  |
| 416 | 1.174 | 114.0914 | Caprolactam | 1.31 | 0.013 |
| 5 | 8.561 | 195.0878 | Caffeine | 10.76 | 0.024 |
| 125 | 2.397 | 284.0990 | Guanosine | 2.34 | 0.004 |
| 165 | 2.407 | 152.0568 | Guanine | 2.26 | 0.001 |
| 360 | 1.505 | 364.0654 | Guanosine monophosphate | 1.41 | 0.004 |
| 92 | 1.946 | 136.0758 | Benzeneacetamide | 1.90 | 0.018 |
|  |  |  | **Others** |  |  |
| 136 | 0.915 | 505.1769 | Maltotriose | 1.96 | 0.000 |
| 276 | 0.833 | 180.0867 | Glucosamine | 1.48 | 0.020 |

Note: Compounds were identified by accurate molecular weight, retention time , MS^2^ and metabolomics database simultaneously

**Table S2.** Correlation analysis between the metabolites from nontargeted analysis and black tea at different fermentation temperatures (or IC_50_ values of α-amylase or IC_50_ values of α-glucosidase)

| Compounds | VIP | α-Glucosidase IC_50_ | α-Amylase IC_50_ |
| --- | --- | --- | --- |
|  |  | *R* | *R* |
| **Catechins** |  |  |  |
| EGCG | 5.30 | 0.95*** | 0.69* |
| EGC | 4.22 | 0.95*** | 0.55 |
| GCG | 2.52 | 0.97*** | 0.68* |
| C | 1.77 | 0.96*** | 0.68* |
| GC | 2.34 | 0.86** | 0.51 |
| CG | 1.14 | 0.92*** | 0.66 |
| Epigallocatechin 3-O-(3-O-methyl)gallate | 1.25 | 0.93*** | 0.73* |
| **Dimeric catechins** |  |  |  |
| EC-(4β→8)-EGC | 1.99 | 0.80** | 0.55 |
| Theasinensin B | 3.12 | 0.93*** | 0.59 |
| Procyanidin B2 | 2.50 | 0.93*** | 0.60 |
| Theasinensin C | 2.73 | 0.85** | 0.34 |
| Theasinensin A | 1.60 | 0.91*** | 0.80** |
| Theaflavin | 1.66 | 0.83** | 0.38 |
| Procyanidin B1 | 1.27 | 0.90*** | 0.60 |
| EC-(4β→8)-EGCG | 1.22 | 0.96*** | 0.83** |
| **Flavonol glycosides and flavone glycosides** |  |  |  |
| Myricetin 3-O-glucoside | 5.64 | 0.96*** | 0.59 |
| Myricetin-3-O-galactoside | 2.60 | 0.97*** | 0.66 |
| **Amino acids** |  |  |  |
| L-Tyrosine | 1.96 | 0.82** | 0.61 |
| L-(+)-Leucine | 5.59 | -0.92*** | -0.77* |
| L-(+)-Valine | 2.53 | -0.69* | -0.88** |
| Oxidized glutathione | 1.93 | 0.76* | 0.22 |
| **Orgnic acids and aldehydes** |  |  |  |
| Theogallin | 5.64 | 0.46 | 0.75* |
| Gallic acid | 2.60 | 0.80** | 0.55 |
| 2-Hydroxycinnamic acid | 1.99 | 0.73* | 0.81** |
| 4-Hydroxybenzoic acid | 2.49 | 0.95*** | 0.77* |
| 4-Hydroxybenzaldehyde | 1.56 | 0.94*** | 0.64 |
| cis,cis-Muconic acid | 2.90 | -0.56 | -0.04 |
| 2,5-Dihydroxybenzaldehyde | 1.47 | 0.98*** | 0.73* |
| **Alkaloids** |  |  |  |
| Caprolactam | 1.31 | -0.89** | -0.41 |
| Caffeine | 10.76 | -0.77* | -0.55 |
| Guanosine | 2.34 | -0.78* | -0.30 |
| Guanine | 2.26 | -0.85** | -0.41 |
| Guanosine monophosphate | 1.41 | -0.85** | -0.45 |
| Benzeneacetamide | 1.90 | 0.89** | 0.63 |
| **Others** |  |  |  |
| Maltotriose | 1.96 | 0.88** | 0.46 |
| Glucosamine | 1.48 | 0.05 | -0.32 |

Note: * indicates *P*＜0.05; ** indicates *P*＜0.01;*** indicates *P*＜0.001


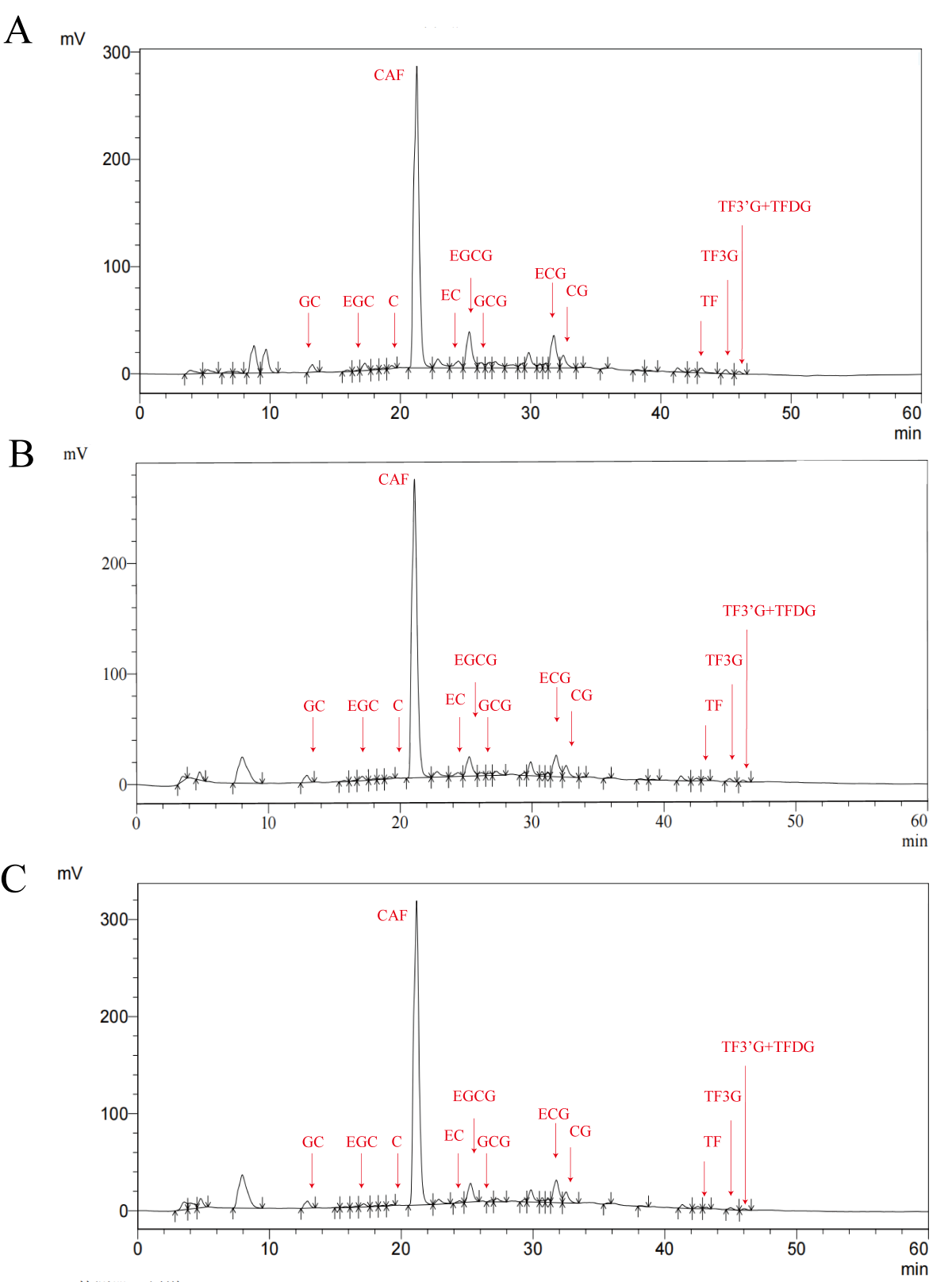


Figure S.1.High performance liquid chromatography of catechins and theaflavins in Jinxuan black tea at fermentation temperature of 20℃(a),fermentation temperature of 25℃(b),fermentation temperature of 30℃(c).
